# Polysaccharide Accumulation and Antioxidant Activity in Space-Mutated *Spirulina platensis* H11 Under Seawater Versus Freshwater Cultivation

**DOI:** 10.3390/md24080272

**Published:** 2026-08-05

**Authors:** Weinan Wang, Tao Li, Hualian Wu, Houbo Wu, Yingyun Cui, Hui Deng, Wenzhou Xiang, Chuanmao Li

**Affiliations:** 1 State Key Laboratory of Breeding Biotechnology and Sustainable Aquaculture, Guangdong Key Laboratory of Marine Materia Medica, South China Sea Institute of Oceanology, Chinese Academy of Sciences, Guangzhou 510301, China; wangweinan0220@163.com (W.W.); taoli@scsio.ac.cn (T.L.); hlwu@scsio.ac.cn (H.W.); wuhoubo@scsio.ac.cn (H.W.); 2 University of Chinese Academy of Sciences, Beijing 101408, China; 3 Guangzhou Keneng Cosmetic Scientific Research Co., Ltd., Guangzhou 510800, China; cuiyy@danzi.cn (Y.C.); denghb@danzi.cn (H.D.)

**Keywords:** *Spirulina platensis*, space-flight environment, polysaccharide, seawater, fresh water, physicochemical characterization, antioxidant activity

## Abstract

As a microalga of significant economic value, the polysaccharide synthesis efficiency and bioactivity of *Spirulina platensis* are significantly influenced by the culture condition. In this study, *S*. *platensis* H11 obtained by space mutagenesis, was systematically compared to the growth characteristics, polysaccharide properties, and antioxidant activities between seawater and freshwater culture conditions. The results showed that the biomass concentration (8.87 g L^−1^) and polysaccharide yield (5.46 g L^−1^) of the strain in the seawater condition were increased by 16.1% and 14.7%, respectively, compared with that in the freshwater condition. Seawater-derived polysaccharides (SSPS) featured an α-configuration glucan backbone and exhibited higher molecular weight (466.19 kDa) and an additional β-configured anomeric signal compared with FSPS. Despite having lower contents of sulfate groups (0.56% DW) and glucuronides (4.92% DW) by 39.3% and 33.5%, respectively, compared to FSPS (0.78% sulfate group, 6.57% glucuronide), SSPS exhibited significantly enhanced hydroxyl radical-scavenging activity, demonstrated by a decreased IC_50_ value of 2.53 mg mL^−1^ as opposed to 3.07 mg mL^−1^. The present study confirms that seawater cultivation is an effective strategy for inducing the synthesis of highly active polysaccharides in space mutant strains, and provides a theoretical basis for the targeted production of polysaccharides in *S*. *platensis* and their functional and precise application.

## 1. Introduction

*Spirulina platensis* (*Arthrospira platensis*) is a member of the Cyanobacteria (Oscillatoriaceae) family that exhibits remarkable tolerance to fluctuations in salinity, pH, and temperature [1]. *Spirulina* polysaccharides (SPS), the main form of carbohydrates in *Spirulina*, are natural bioactive macromolecules with a complex structure that are usually used to build cell wall and serve as energy reserves [2]. Due to the biological activities such as immunomodulation, antiviral activity, improvement of gut microbiota, and antioxidant activity, SPS have become the focus of research in the field of functional food and cosmetics [3,4,5,6].

In the traditional freshwater culture mode, the polysaccharide content of *S*. *platensis* only constitutes 10–15% of the dry weight, which restricts its industrial applications [7]. Environmental stress, especially salt stress, has emerged as a useful strategy for enhancing production of polysaccharides. Bezerra et al. found that cultivation of *Spirulina* sp. LEB 18 in seawater without nutrient supplementation increased carbohydrate content and productivity by 203% and 52%, respectively [8]. Similarly, *S*. *platensis* cultured in seawater showed higher carbohydrate content than that in freshwater [9]. However, most studies have treated seawater merely as a low-cost medium, focusing on biomass carbohydrate accumulation or pigment production rather than systematic characterization of the isolated polysaccharides. Moreover, comparisons of polysaccharide yield, structural features, and bioactivities between seawater and freshwater cultures remain limited.

The previous studies showed that salinity stress not only promoted the accumulation of polysaccharides but also significantly altered the structure and enhanced bioactivities. Mishra and Jha found that the amount of four monosaccharides (galactose, glucose, xylose, and fructose) in *Dunaliella salina* polysaccharides increased at 3 M salinity [10]. Under 0.3 M NaCl stress conditions, the proportion of fucose, glucose, and glucuronic acid decreased, while the proportion of galactose, mannose, and xylose increased in the exopolysaccharides of *Nostoc* sp. HK–01 [11]. Crucially, structural modifications induced by salinity are often closely associated with enhanced biological activity. Li et al. demonstrated that the extracellular polysaccharides of *Porphyridium purpureum* synthesized under high salinity conditions had higher hydroxyl radical-scavenging activity compared to the low salinity conditions [12]. Similarly, polysaccharides from *Porphyridium* sp. and *Porphyridium aerugineum* cultured in high concentrations of each salt (including NaCl/KCl/MgCl_2_/CaCl_2_/SrCl_2_) exhibited better antioxidant activities in comparison to the low salt concentrations [13]. These studies show that salinity can alter polysaccharide composition and antioxidant activity. However, the effects of seawater cultivation on the structural characteristics and bioactivities of *Spirulina* polysaccharides remain to be elucidated.

Recently, space mutation breeding has emerged as a novel method to induce genetic mutations in plants, utilizing microgravity, high-energy ion radiation, and other unusual space environments [14]. Previous studies conducted in our laboratory have confirmed the genetic stability and elevated polysaccharide production in the space-mutagenized *S*. *platensis* SCSIO-44012 H11 [15,16]. In the present study, the differences in structural characteristics, physicochemical properties and antioxidant activities of polysaccharides of *S*. *platensis* H11 under seawater and freshwater were investigated, providing a guidance for the production of highly active polysaccharides in *S*. *platensis* H11.

## 2. Results

### 2.1. The Growth and Polysaccharide Production of S. platensis H11 in Seawater and Freshwater

**Figure 1:**
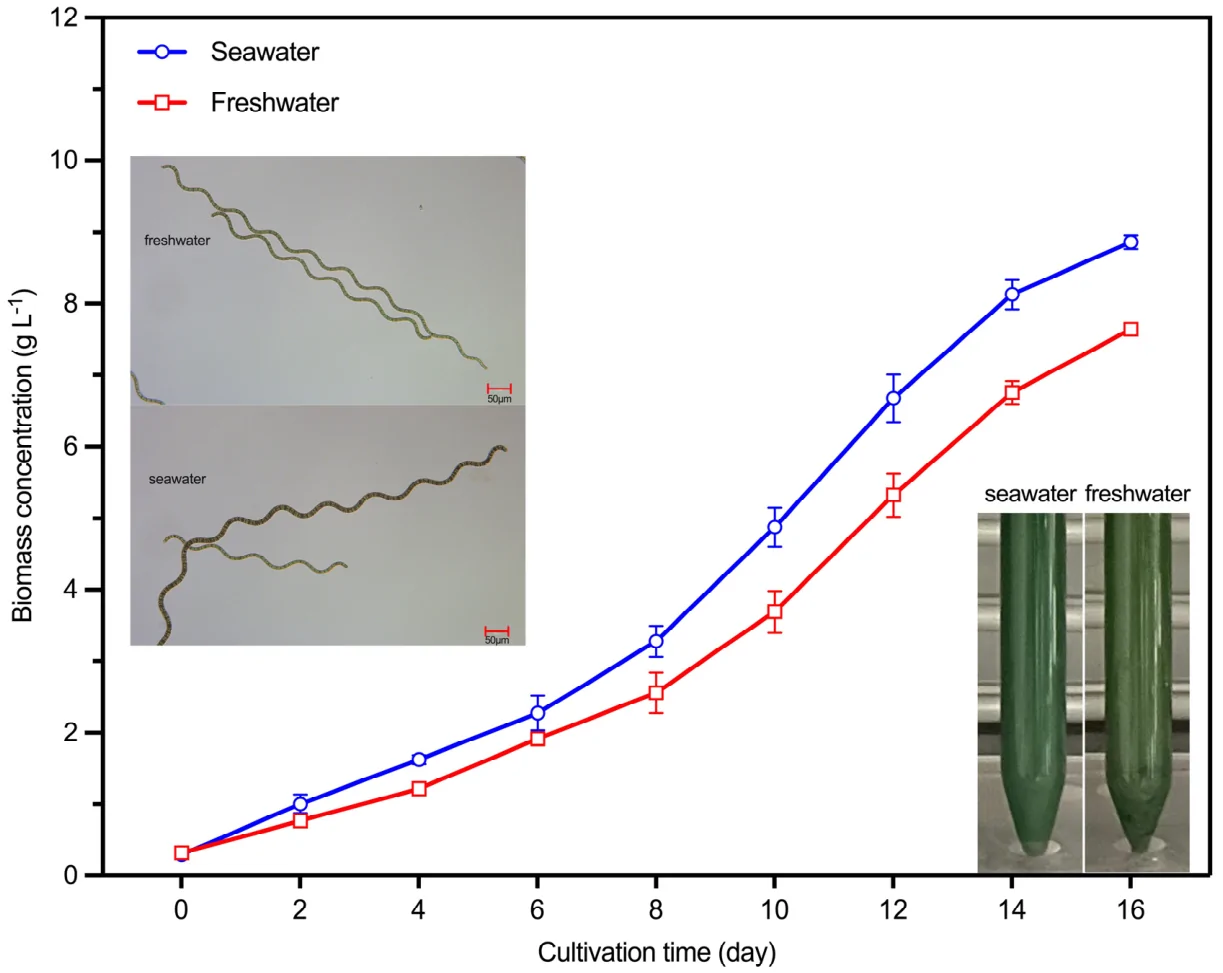
Biomass concentration of *S. platensis* H11 cultured in seawater and freshwater (*n* = 3; data: mean ± SD).

The growth of *S*. *platensis* in seawater and freshwater was described in biomass concentration (Figure 1). The biomass concentration of *S*. *platensis* cultured in seawater was up to 8.87 g L^−1^, which was significantly higher (*p* < 0.05) than that in freshwater (7.64 g L^−1^). As shown in Table 1, the average specific growth rate did not differ significantly between the seawater treatment (0.21 ± 0.03 d^−1^) and the freshwater treatment (0.20 ± 0.03 d^−1^; *p* > 0.05). However, the maximum specific growth rate was significantly higher in the seawater treatment (0.61 ± 0.04 d^−1^) than in the freshwater treatment (0.44 ± 0.03 d^−1^; *p* < 0.05). Similarly, biomass productivity was significantly higher in the seawater treatment (0.54 ± 0.05 g L^−1^ d^−1^) than in the freshwater treatment (0.46 ± 0.01 g L^−1^ d^−1^; *p* < 0.05). A significant difference in the color of the two treatments was observed at the end of the cultivation, with the seawater culture showing a bluish-green color, and the freshwater culture showing a yellowish-green color.

**Figure 2:**
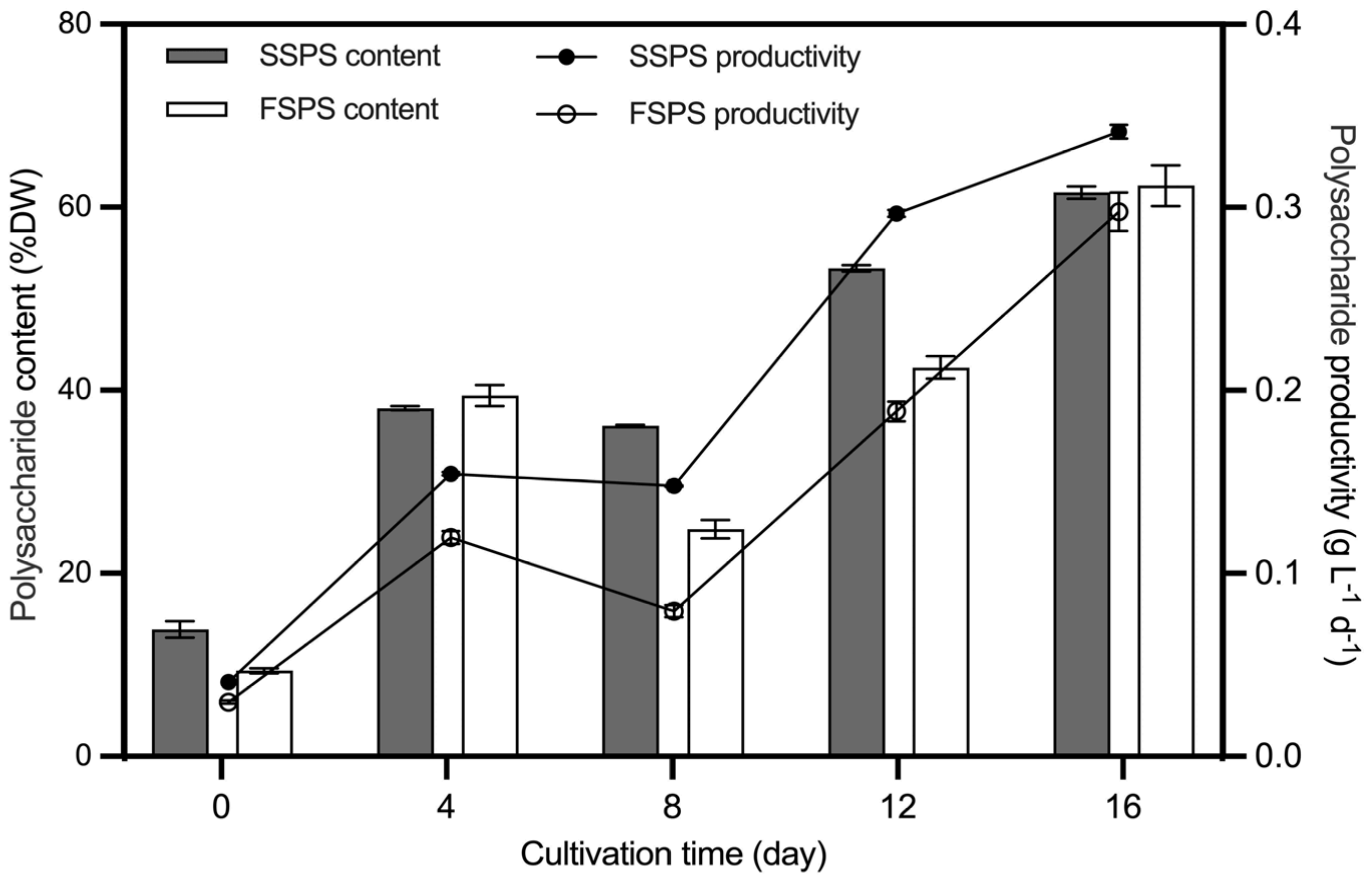
The content and productivity of polysaccharides of *S*. *platensis* H11 in seawater and freshwater (SSPS: seawater *Spirulina* polysaccharides, FSPS: freshwater *Spirulina* polysaccharides) (*n* = 3; data: mean ± SD).

The polysaccharide content and polysaccharide productivity were employed to evaluate the capacity of *S*. *platensis* H11 to accumulate polysaccharides in seawater and freshwater. The polysaccharide content of *S*. *platensis* H11 cultured in seawater exhibited a consistently higher value than that of the freshwater by day 12 (Figure 2). On the 16th day, there was no significant difference in the polysaccharide content, which was 61.61% DW and 62.35% DW (*p* > 0.05), respectively. The polysaccharide productivity in seawater was 0.34 g L^−1^ d^−1^, which was significantly higher than that in freshwater (*p* < 0.05).

### 2.2. The Preparation and Morphology of Polysaccharides from S. platensis H11

**Figure 3:**
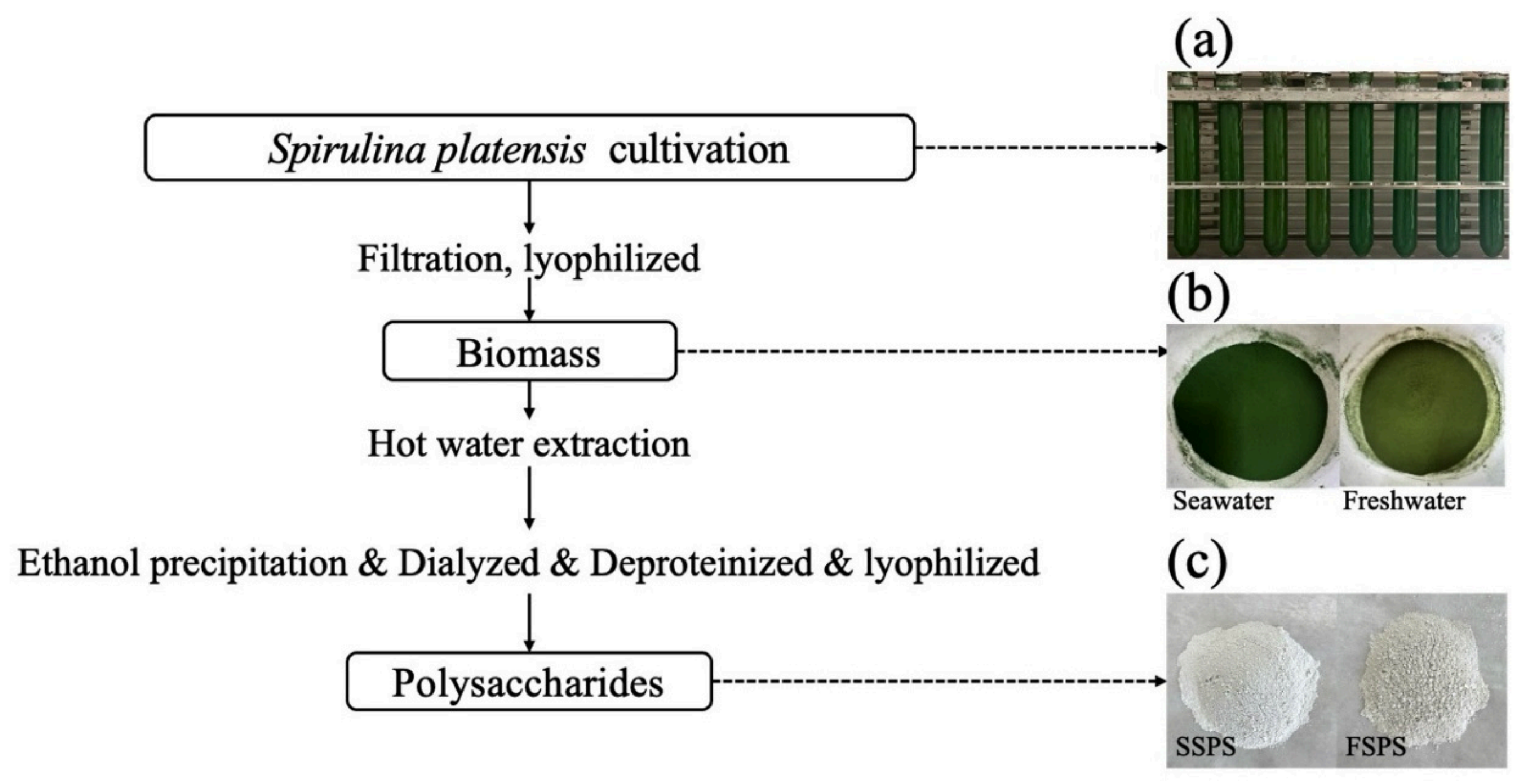
Preparation and purification of *S*. *platensis* H11 polysaccharides. (**a**) The culture of *S. platensis*, (**b**) the freeze-dried microalgal powder, (**c**) polysaccharides from seawater and freshwater (SSPS and FSPS).

**Figure 4:**
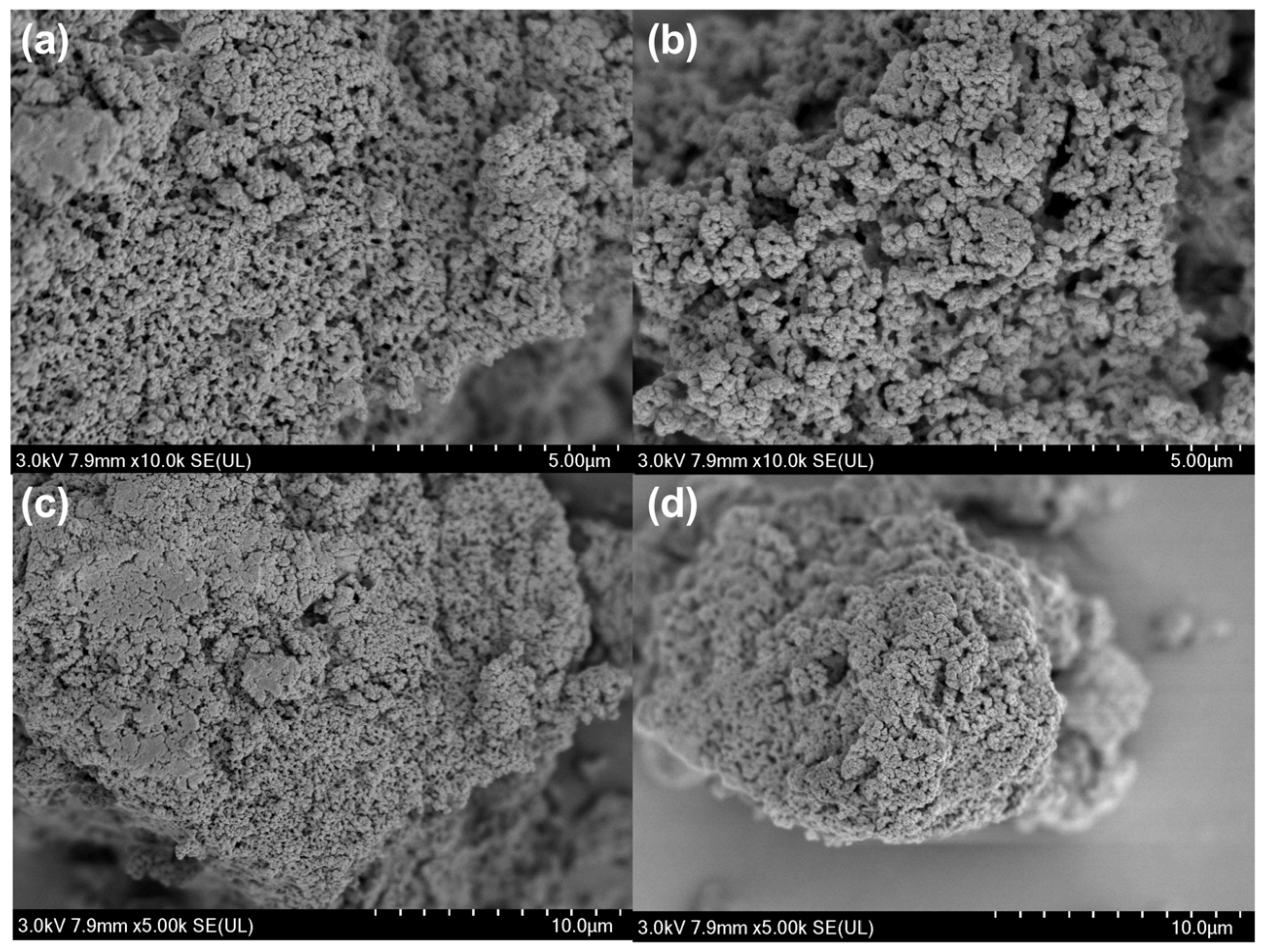
Microstructure of *Spirulina* polysaccharides under scanning electron microscopy ((**a**): SSPS, ×10,000; (**b**): FSPS, ×10,000; (**c**): SSPS, ×5000; (**d**): FSPS, ×5000) (SSPS: seawater *Spirulina* polysaccharides, FSPS: freshwater *Spirulina* polysaccharides).

SSPS and FSPS were prepared by the procedure shown in Figure 3. The apparent morphology of SPS exhibited a powder form with different colors. SSPS were white and FSPS were greyish-white with more obvious particles. Scanning electron microscopy (SEM) revealed that both samples exhibited a granular surface structure, with SSPS displaying larger pore grains and pores and a more irregular and porous surface morphology (Figure 4).

### 2.3. Differential Analysis of the Physicochemical Properties of Spirulina Polysaccharides H11

**Figure 5:**
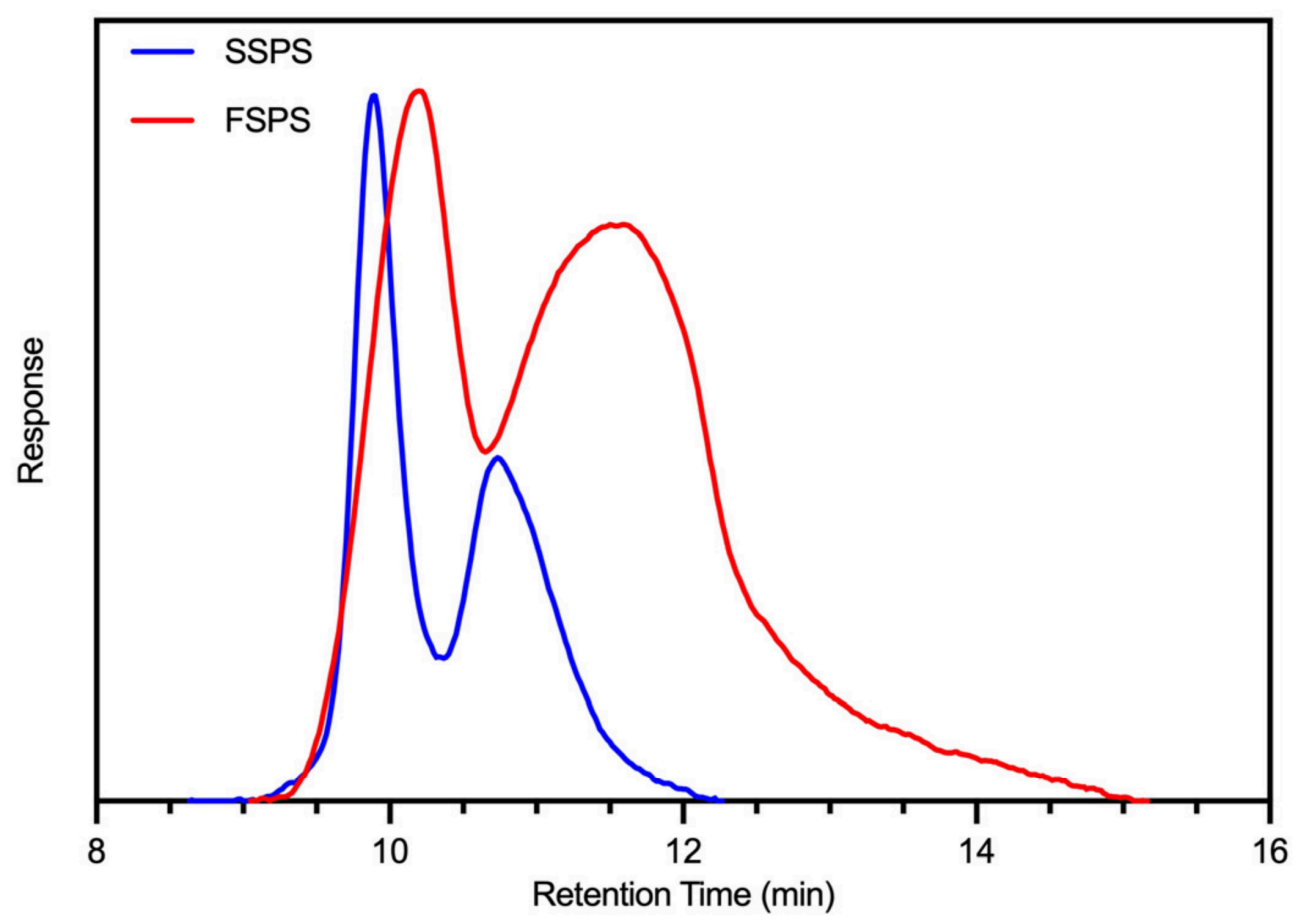
Molecular weight outflow curve of *Spirulina* polysaccharides (SSPS: seawater *Spirulina* polysaccharides, FSPS: freshwater *Spirulina* polysaccharides).

The molecular weights of SSPS and FSPS were determined by using gel permeation chromatography (GPC). Both SSPS and FSPS exhibited bimodal GPC profiles, suggesting the presence of two molecular-weight populations (Figure 5). The molecular weight of the highest peak (Mp) of the dominant peak was 650.73 kDa for SSPS and 490.92 kDa for FSPS. Based on integration of the entire molecular-weight distribution, SSPS exhibited a higher weight average molecular weight (Mw) than FSPS (466.19 versus 276.97 kDa) and a lower polydispersity coefficient (1.28 versus 2.36), indicating a higher overall molecular weight and a narrower molecular-weight distribution. The results demonstrated that the molecular weight distribution of SSPS was more intensive than that of FSPS, suggesting that the composition of its polysaccharide species was simpler than that of FSPS (Table 2).

The total carbohydrate, proteins, uronic acids and sulphates of SSPS and FSPS were determined (Table 3). SSPS possessed a higher total carbohydrate content of up to 88.79% (*p* < 0.05), while FSPS had higher protein, uronic acid and sulphate contents of 6.69%, 6.57% and 0.78%, respectively (*p* < 0.05). After several rounds of deproteinization, proteins were still detected in the *Spirulina* polysaccharides, indicating that a certain amount of protein covalently bound to the polysaccharides.

**Figure 6:**
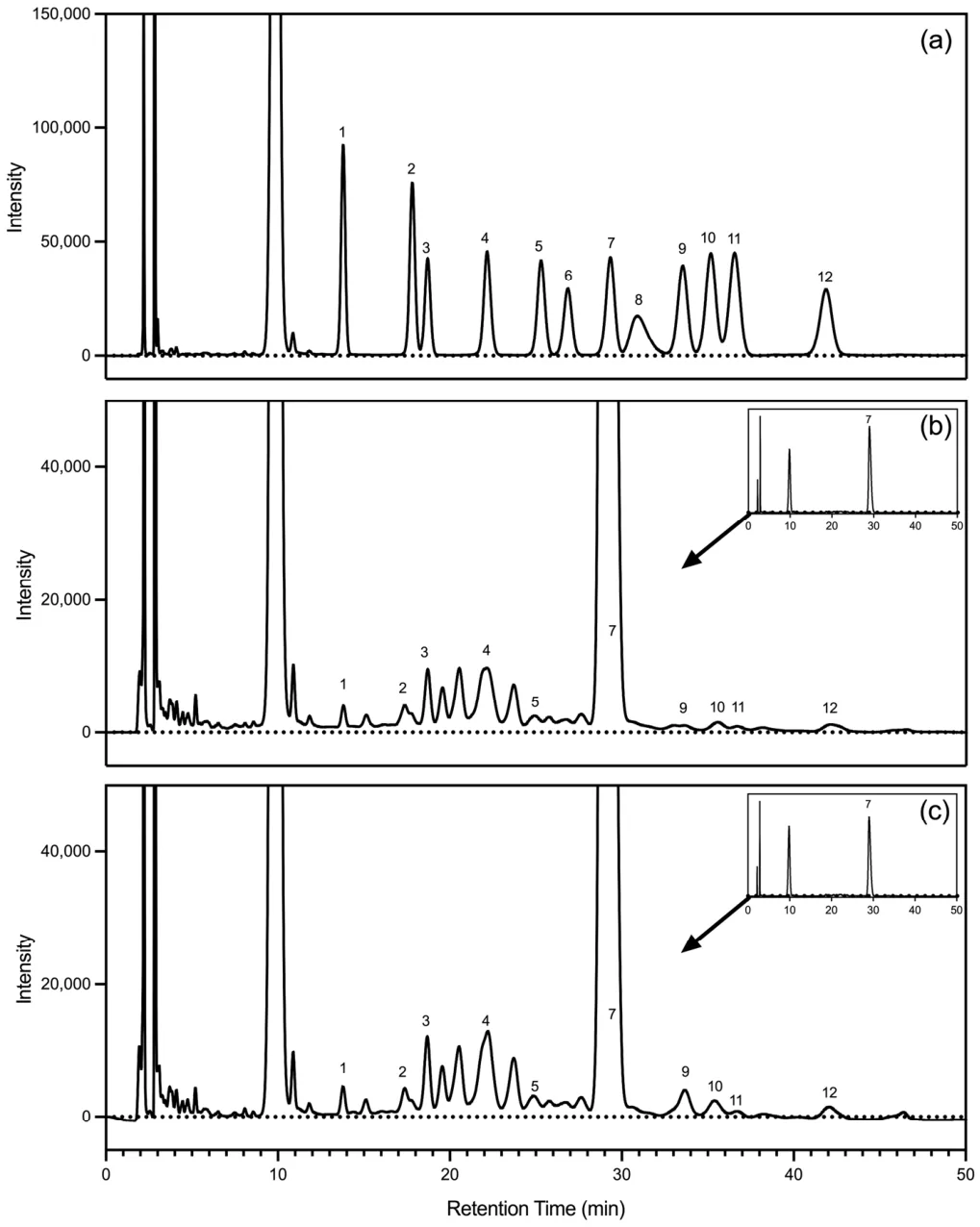
High-performance liquid chromatography profiles of (**a**) mixed standard monosaccharides, (**b**) SSPS, (**c**) FSPS (SSPS: seawater *Spirulina* polysaccharides, FSPS: freshwater *Spirulina* polysaccharides). The peaks in chromatography profile (**a**) from left to right are as follows: (1) mannose; (2) ribose; (3) rhamnose; (4) glucuronic acid; (5) galacturonic acid; (6) N-acetyl-glucosamine; (7) glucose; (8) N-acetyl-galactosamine; (9) galactose; (10) xylose; (11) arabinose; (12) fucose.

The monosaccharide composition of the derivatized polysaccharides was determined by high-performance liquid chromatography (HPLC) (Figure 6). Both fractions were strongly glucose-dominant, with glucose accounting for 95.12% and 92.97%, respectively (*p* < 0.05). The other eight monosaccharides were present only as minor or trace components, collectively accounting for 4.88% of SSPS and 7.03% of FSPS. The results showed that *Spirulina* polysaccharides are a kind of macromolecular heteropolysaccharide mainly composed of glucose, with a minor proportion of glucuronic acid and sulfate groups. Compared to FSPS, SSPS displayed a more consistent composition with a higher molecular weight, reducing subsequent isolation and purification costs.

**Figure 7:**
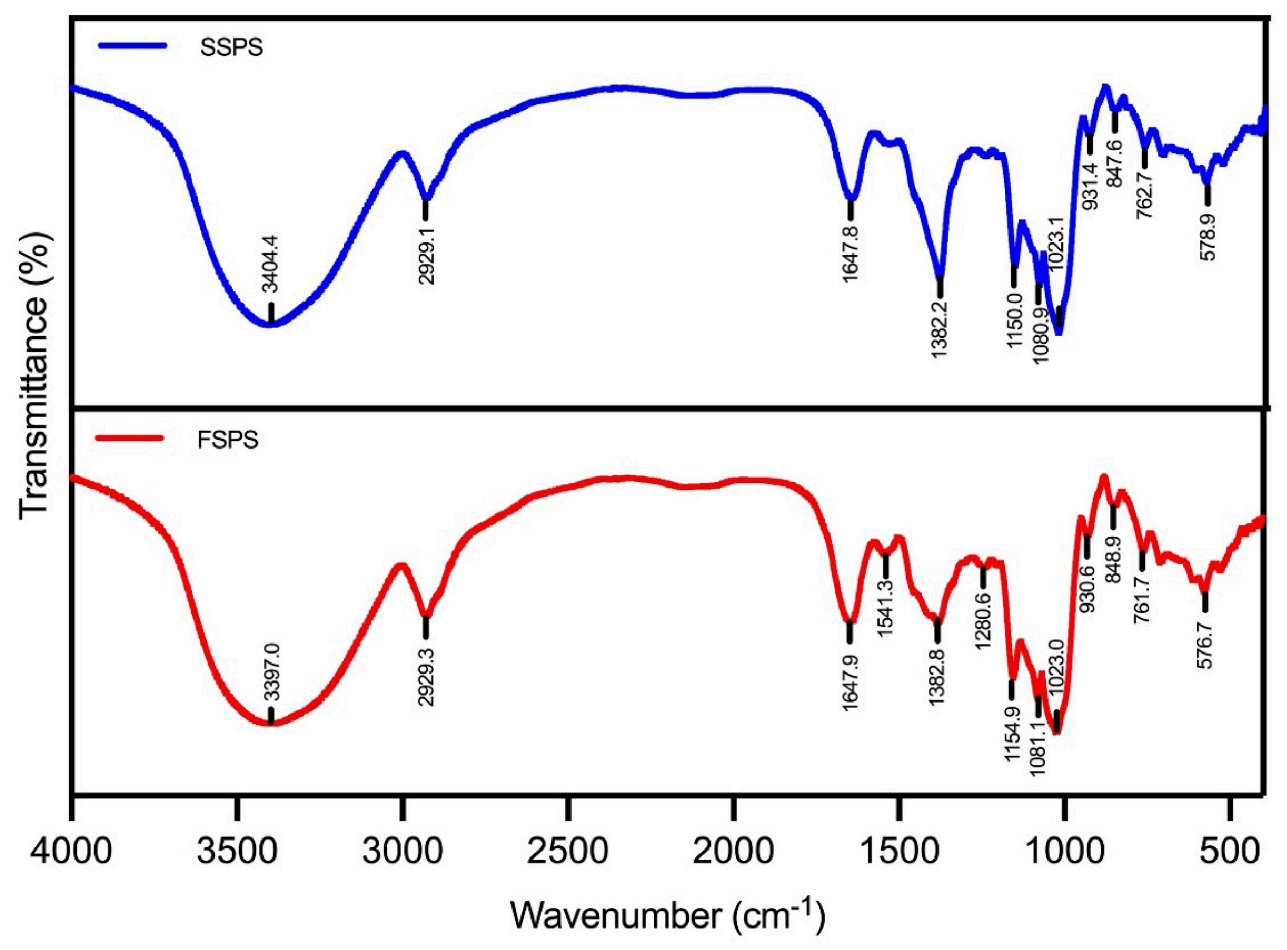
Fourier-transform infrared absorption spectra of *Spirulina* polysaccharides (SSPS: seawater *Spirulina* polysaccharides, FSPS: freshwater *Spirulina* polysaccharides).

The Fourier-transform infrared (FT-IR) absorption spectra of SSPS and FSPS in the range of 4000–400 cm^−1^ were shown in Figure 7. The broad absorption peaks at 3397–3404 cm^−1^ were attributed to the stretching vibration of the hydroxyl group (-OH). The weak absorption peak at 2929 cm^−1^ may be associated with the stretching vibration of the C-H bond on the sugar ring, combined with the absorption peak at 1382 cm^−1^ caused by the bending vibration of the C-H bond, which is the characteristic infrared absorption peak of polysaccharides [17]. The absorption peak at 1647 cm^−1^ is generated by the C=O stretching vibration in the amide bond (-CONH-) of amide I, which confirms the existence of proteins in *Spirulina* polysaccharides [18]. The peaks observed near 1426 and 1541 cm^−1^ in FSPS were attributed to the stretching vibration of the -CHO and -C=O bonds of the -COOH group in the uronic acid, which confirmed the presence of uronic acid in polysaccharides. In contrast, the characteristic peaks of uronic acid in SSPS were not obvious, suggesting that the content of uronic acid in the SSPS was much lower, which was similar to the results of the chemical composition determination [19]. The broad bands at 1081 and 1023 cm^−1^, induced by C-OH stretching vibrations and C-O stretching vibrations in C-O-C, indicate that Spirulina polysaccharides contain sugar residues in the pyranose configuration [20]. Meanwhile, the absorption band at 931 cm^−1^ and 844 cm^−1^ was attributed to the presence of *α*-type glycosidic bond [21]. The results of infrared spectroscopy showed that both SSPS and FSPS had typical absorption peaks of polysaccharides, with the predominant presence of the *α*-configuration pyranose ring.

**Figure 8:**
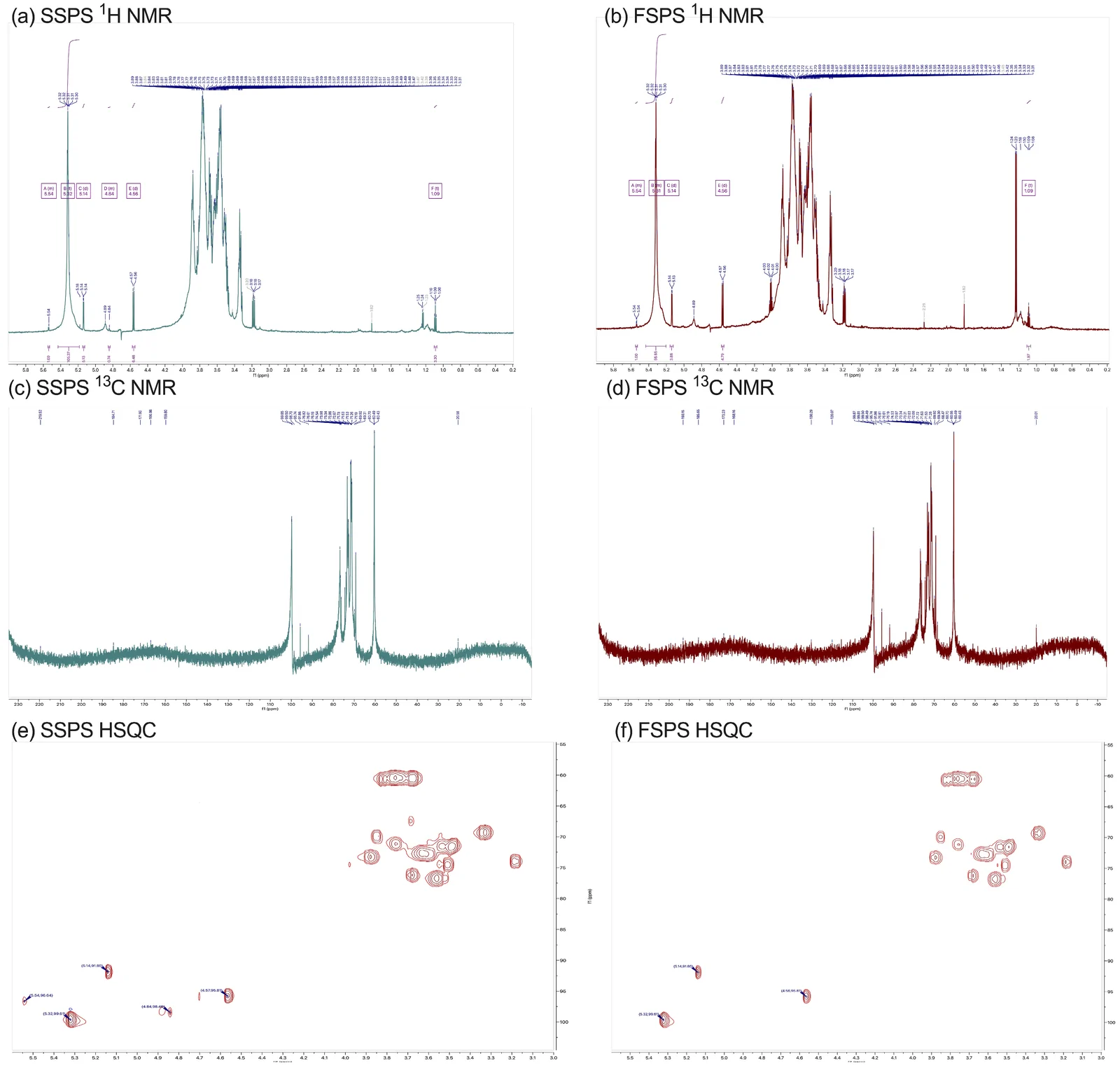
Nuclear magnetic resonance spectroscopy of SPSS and FSPS. (**a**,**b**) 1H NMR, (**c**,**d**) 13C NMR, (**e**,**f**) HSQC (SSPS: seawater *Spirulina* polysaccharides, FSPS: freshwater *Spirulina* polysaccharides).

The 1D and 2D NMR spectra of SSPS and FSPS are shown in Figure 8. In the ^1^H nuclear magnetic resonance (NMR) spectrum, the chemical shift range of 4.4–5.6 ppm corresponds to the anomeric proton region. For SSPS, five anomeric protons labeled A, B, C, D, and E were observed at 5.54 (d, J = 3.95 Hz), 5.32 (d, J = 3.93 Hz), 5.14 (d, J = 3.81 Hz), 4.84 (d, J = 8.08 Hz), and 4.56 (d, J = 3.59 Hz) ppm, respectively (Figure 8a). The C1/H1 cross-peaks identified in the HSQC spectrum (Figure 8e) allowed assignment of the corresponding anomeric carbon signals in the ^13^C NMR spectrum (Figure 8c) to 96.64, 99.61, 91.85, 98.46, and 95.81 ppm, confirming the presence of five distinct sugar residues. The anomeric proton and carbon regions for *α*-configured residues were typically observed at 5.1–5.8 ppm and 98–103 ppm, respectively, whereas *β*-configured residues corresponded to 4.3–4.8 ppm and 103–106 ppm. These data indicated that residues B (*δH* 5.32, *δC* 99.61), C (*δH* 5.14, *δC* 91.85), and E (*δH* 4.56, *δC* 95.81) were *α*-glucopyranose residues. Additionally, the F residue observed at 1.09 ppm (t, J = 7.07 Hz) in SSPS suggested the presence of a methyl group. Combined with the anomeric proton shift of residue A (*δH* 5.54 ppm) and its corresponding carbon signal (*δC* 96.64 ppm), residue A was tentatively identified as an *α*-L-rhamnopyranose residue. The anomeric proton shift of residue D (*δH* 4.84 ppm, J = 8.08 Hz) aligned with the characteristics of a *β*-configured sugar residue. Integration of monosaccharide composition analysis confirmed that SSPS comprised three *α*-glucopyranose residues, one *β*-glucopyranose residue, and one *α*-rhamnopyranose residue. In FSPS, four anomeric protons labeled A, B, C, and E were detected at 5.54 (d, J = 4.19 Hz), 5.32 (d, J = 3.91 Hz), 5.14 (d, J = 3.79 Hz), and 4.56 (d, J = 8.00 Hz) ppm, respectively. The NMR spectra of FSPS reflected the absence of the *β*-constitutive glycosidic bond represented at residue D in freshwater-cultured *Spirulina* compared to SSPS.

### 2.4. Antioxidant Activities Evaluation of Spirulina Polysaccharides

**Figure 9:**
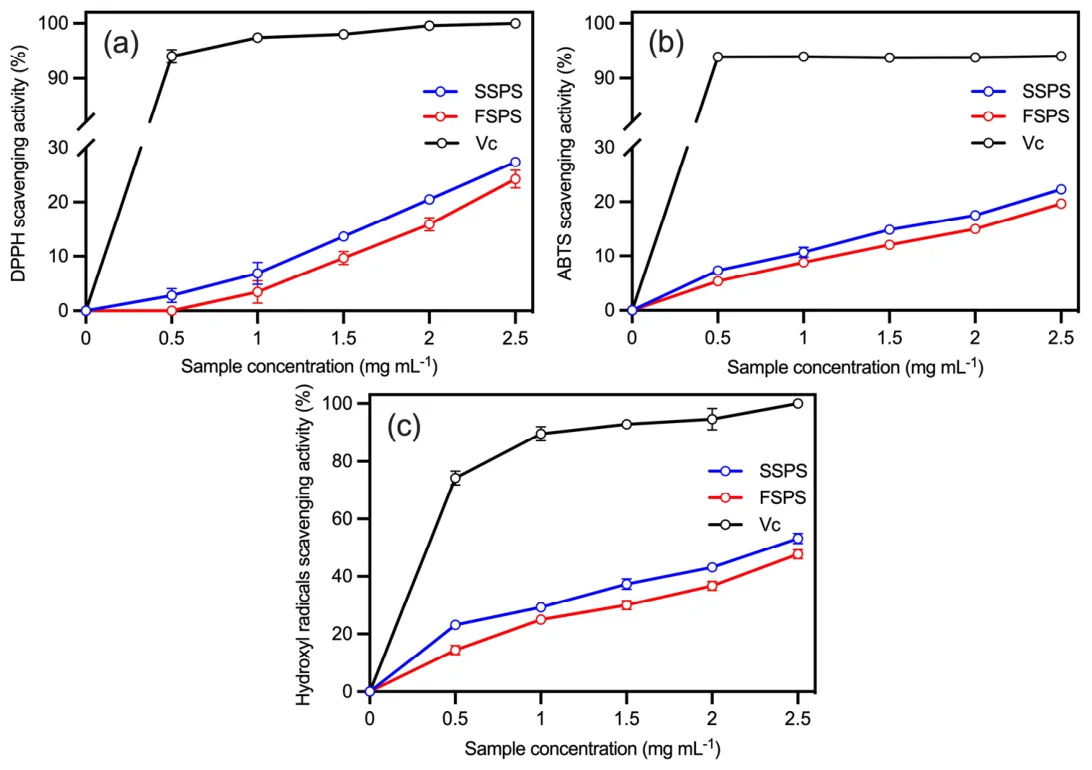
Antioxidant activities of *Spirulina* polysaccharides. (**a**) Scavenging activity against DPPH radicals; (**b**) ABTS radicals; (**c**) hydroxyl radicals. (*n* = 3; data: mean ± SD).

The difference in antioxidant activity between SSPS and FSPS was compared by measuring the scavenging activity against three free radicals (DPPH, ABTS, and hydroxyl radicals), and the results are shown in Figure 9.

There was a dose-dependent effect between the polysaccharide concentration and the DPPH scavenging effect, as shown in Figure 9a. SSPS possessed better DPPH scavenging capacity, with the lowest DPPH scavenging effect observed at 0.5 mg mL^−1^, which was 2.85% for SSPS, whereas FSPS exhibited no scavenging activity. Since neither sample achieved 50% scavenging within the tested concentration range, the IC_50_ values for SSPS and FSPS (4.10 and 4.43 mg mL^−1^, respectively) were extrapolated from the fitted dose–response curves (Table 4).

The scavenging capacities of SSPS and FSPS against ABTS radicals are characterized in Figure 9b. The scavenging rate of ABTS by SSPS and FSPS increased in a dose-dependent manner across the concentration range of 0.5 to 2.5 mg mL^−1^. At a concentration of 2.5 mg mL^−1^, the scavenging effect of SSPS was superior to that of FSPS, with the maximum scavenging activity reaching 22.27% and 19.67%, respectively (*p* < 0.05). Because 50% scavenging was not reached within the tested range, the IC_50_ values determined using GraphPad Prism 10 (10.92 mg mL^−1^ for SSPS and 11.36 mg mL^−1^ for FSPS) are approximate and should be interpreted with caution.

The hydroxyl radical-scavenging capacity of SSPS and FSPS is depicted in the concentration range of 0.5–2.5 mg mL^−1^ in Figure 9c. The maximum scavenging activities of SSPS and FPSP were 53.26% and 47.78% at a sample concentration of 2.5 mg mL^−1^, with IC_50_ of 2.53 mg mL^−1^ and 3.07 mg mL^−1^, respectively (*p* < 0.05) (Table 4).

SSPS exhibited stronger radical-scavenging activity than FSPS across all three assays, with the largest difference observed in the hydroxyl radical system. At 2.5 mg mL^−1^, SSPS and FSPS scavenged 53.26% and 47.78% of hydroxyl radicals, respectively, with SSPS reaching more than 50% for the activity of ascorbic acid at this concentration. In contrast, scavenging rates against DPPH and ABTS radicals did not exceed 30% for either polysaccharide, even at the highest concentration tested. Both polysaccharides were significantly less active than ascorbic acid (*p* < 0.05).

## 3. Discussion

*S*. *platensis* H11, derived from space mutagenesis, showed a rapid growth rate and a high polysaccharide content in our previous study [16]. Although elevated salinity can inhibit the growth of many freshwater microalgae [22], *S*. *platensis* H11 achieved a higher final biomass concentration in the artificial seawater-based medium than in the freshwater-based medium, indicating its tolerance to the overall ionic environment of artificial seawater. Polysaccharide content was higher in seawater cultures during the early cultivation phase, but this difference was absent at harvest. Thus, its higher volumetric polysaccharide productivity likely resulted mainly from greater biomass production rather than enhanced cellular polysaccharide accumulation. Previous studies have reported elevated carbohydrate concentrations in *S*. *platensis* under NaCl stress, ranging from 20.39–23.36% DW at 200–400 mM NaCl [23] to 20.6% DW at 1.03 M NaCl [24]. This result corroborates the established role of carbohydrates as intracellular osmolytes in maintaining osmotic homeostasis under salt stress [25]. However, while both media were supplemented with identical concentrations of nutrients from the modified Zarrouk formulation, the artificial seawater additionally contributed major ions and trace elements. The observed differences in growth and polysaccharide production therefore likely reflect the combined effects of salinity, ionic strength, and ionic composition, rather than salinity alone. Further studies employing chemically defined media with independently controlled salinity and individual ion concentrations are required to disentangle their respective contributions.

The average specific growth rate, calculated over the entire cultivation period, did not differ significantly between treatments, whereas the maximum specific growth rate and biomass productivity were higher in the artificial seawater treatment. These parameters represent different aspects of growth: the whole-period average specific growth rate reflects the overall relative increase in biomass, the maximum specific growth rate represents the highest interval-specific relative growth rate, and biomass productivity represents the net biomass gain per unit volume and time. Because microalgal growth is phase-dependent, a higher interval-specific growth peak can increase net biomass accumulation without significantly affecting the whole-period average specific growth rate [26,27]. Thus, the artificial seawater medium enhanced peak growth and overall biomass production, but this growth-rate advantage was not sustained throughout cultivation.

SSPS had a higher molecular weight and a narrower molecular weight distribution than that of FSPS. The molecular weights of SSPS and FSPS were 466.19 kDa and 276.97 kDa, respectively. SSPS had a higher total carbohydrate content (up to 88.79%), whereas FSPS contained higher concentrations of uronic acid and sulfate groups, accounting for up to 6.57% and 0.78%, respectively. The typical sufficient concentration of inorganic salt ions in seawater could promote the production of sulfated polysaccharides in microalgae [21]. However, for *S*. *platensis* H11, a higher sulfate concentration in the seawater medium did not enhance the degree of sulfation modification of the polysaccharides. This may be attributed to the fact that the key determinant of polysaccharide sulfation is not the exogenous sulfate concentration but rather the intracellular sulfotransferase activity or sulfate transport efficiency [28]. The primary monosaccharides in SSPS and FSPS were glucoses, accounting for 90% DW of the total polysaccharides. Traces of rhamnose and glucuronic acid made up the remaining ingredients. It was speculated that *S*. *platensis* H11 reached the plateau phase on the 16th day of the culture period, and the algal cells stored α-1,4-linked, α-1,6-branched glucose polymers (glycogen) [29]. The spectra results indicate that the salinity had few effects on the backbone structure of *Spirulina* polysaccharides, while the differences were mainly in the side chains. Compared to SSPS, FSPS had a greater diversity of monosaccharides, as well as a higher content of glucuronic acid and sulfate groups. However, the backbone was linked exclusively by glycosidic linkages in the α-configuration.

The structures of *Spirulina* polysaccharides can be primarily categorized into two types. One type is a glucan characterized by predominantly α-glycosidic bonds and a glycan chain structure of (1→4)-linked-α-D-Glcp, with glucose serving as the major monosaccharide, which is similar to our study [30,31]. The other type is a heteropolysaccharide that possessed a complex monosaccharide composition, primarily rhamnose, galactose, and arabinose, and features a glycosidic linkage of *β*-configuration [6,32,33]. *Spirulina* polysaccharide diversity and structural complexity notwithstanding, the fundamental structure of its polysaccharide backbone appeared to be unaffected by the salinity. Salinity might affect the ratio of different types of polysaccharides.

DPPH and ABTS are widely recognized non-physiological free radicals, providing an indication of the ability of natural products to scavenge free radicals [34]. Hydroxyl radicals are the most common reactive oxygen species within living organisms. High levels of peroxidation subsequently leads to lipid peroxidation and oxidative damage to DNA molecules [35]. SSPS demonstrated stronger radical-scavenging activity than FSPS across all three assays, particularly against hydroxyl radicals. At 2.5 mg mL^−1^, SSPS and FSPS achieved 53.26% and 47.78% scavenging, respectively, with SSPS showing a significantly lower IC_50_ (2.53 vs. 3.07 mg mL^−1^; *p* < 0.05). However, both polysaccharides displayed limited activity toward DPPH and ABTS radicals, with the maximum scavenging rates below 30%. Collectively, these findings suggest that the superior antioxidant potential of SSPS is predominantly attributable to its hydroxyl radical-scavenging capacity.

Previous studies found that *Spirulina* polysaccharides showed the high antioxidant activity under different extraction methods and different activity evaluation systems. Wu et al. obtained polysaccharides from *Spirulina platensis* (PSP), including PSP-3b, which was isolated by high-speed counter-current chromatography (HSCCC) with an ethanol–ammonium sulfate aqueous two-phase system (ATPS). The fraction exhibited the strongest ABTS and DPPH radical-scavenging activities with IC_50_ of 199 μg mL^−1^ and 450 μg mL^−1^ [36]. Polysaccharides from *S. platensis* isolated after optimization of ultrasonic treatment by Forouzan Kurd et al. showed up to 78% and 82% scavenging activity against DPPH and hydroxyl radicals, respectively, at a concentration of 300 μg mL^−1^ [4]. However, the effect of culture-medium composition on the antioxidant activity of polysaccharides in *Spirulina* has not been documented. In the present study, the polysaccharides derived from *Spirulina* cultured in seawater displayed enhanced scavenging activity, despite not exhibiting high concentrations of sulfate groups and uronic acid. Wang et al. compared the in vitro antioxidant activities of *Porphyridium cruentum* CCALA 415 and *Porphyridium purpureum* FACHB 806. Although no significant difference in sulfate content was observed between the two *Porphyridium* strains, their antioxidant activities differed markedly. Although sulfate and uronic acid groups may contribute to antioxidant activity, their contents alone did not determine the radical scavenging capacity of polysaccharides. In the present study, SSPS contained less sulfate and uronic acid than FSPS but exhibited stronger hydroxyl radical-scavenging activity. A similar non-linear relationship between sulfate content and antioxidant activity has been reported previously [37,38]. SSPS had a higher molecular weight than FSPS (466.19 vs. 276.97 kDa), and NMR analysis showed an additional β-configured anomeric signal in SSPS. These structural differences might influence chain conformation and the accessibility of functional groups involved in radical scavenging. However, because polysaccharide conformation was not directly determined, the enhanced activity of SSPS could not be attributed to any single structural feature.

## 4. Materials and Methods

### 4.1. Microalgae and Culture Conditions

*S*. *platensis* SCSIO-44012 H11 was derived from space flight inducement through the scientific recoverable satellite Shijian-10, which was carried on board the Science Recovery Satellite Practice 10 launched on 6 April 2016. The strain was subsequently preserved in the South China Sea Institute of Oceanology, Chinese Academy of Sciences (Guangzhou, China).

**Figure 10:**
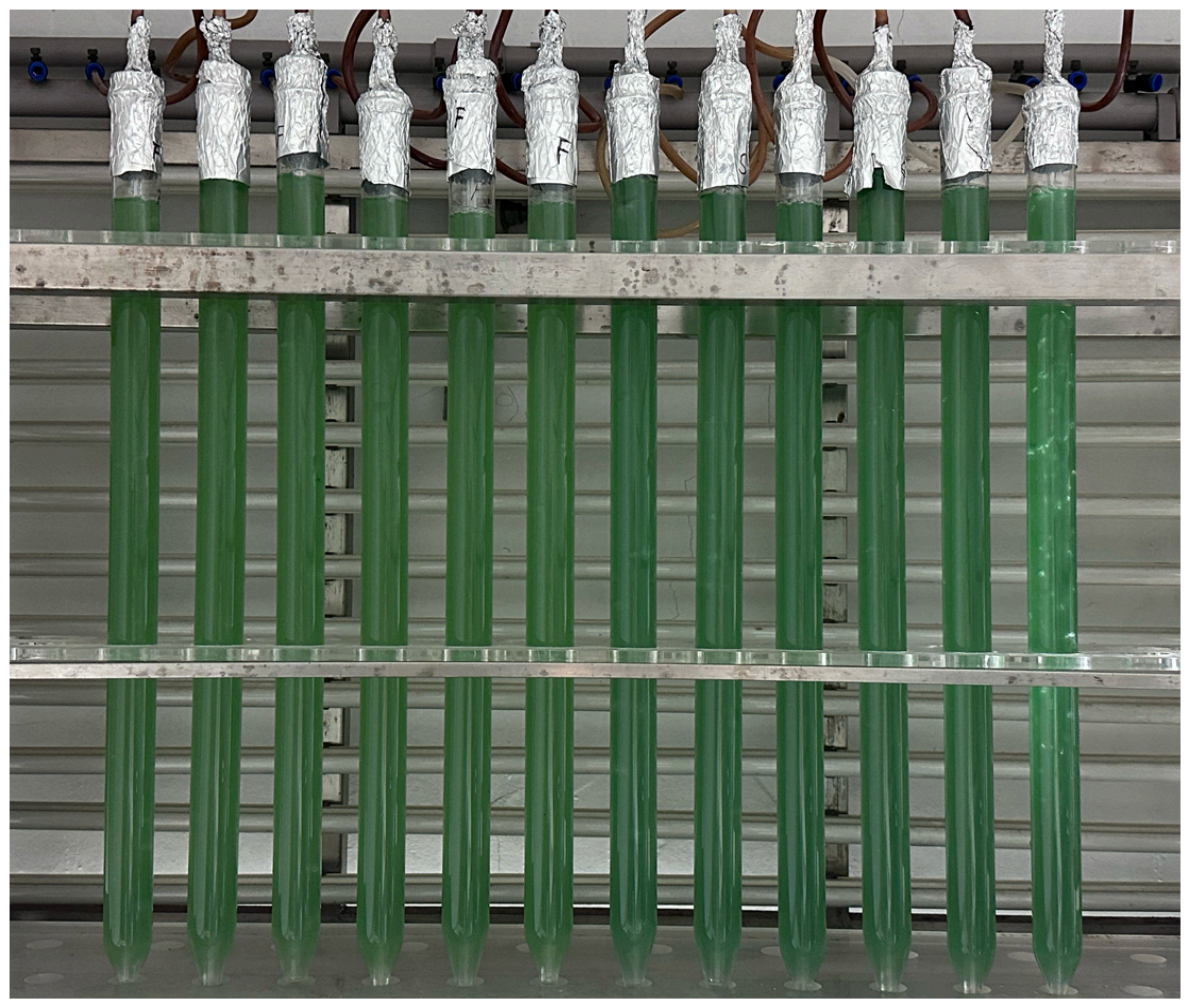
Column photobioreactor system used for seawater and freshwater cultivation of *S*. *platensis*.

It was inoculated in Φ3.0 cm × 60 cm glass column photobioreactors containing 300 mL of modified Zarrouk medium, which contained 29.41 mmol NaNO_3_, 17.09 mmol NaCl, 11.90 mmol NaHCO_3_, 2.87 mmol K_2_HPO_4_, 0.81 mmol MgSO_4_ 7H_2_O, 5.75 mmol K_2_SO_4_, 0.27 mmol CaCl_2_ 2H_2_O, 0.21 mmol Na_2_EDTA 2H_2_O, 35.97 μmol FeSO_4_ 7H_2_O, 0.91 μmol MnCl_2_ 4H_2_O, 0.08 μmol ZnSO_4_ 7H_2_O, 0.02 μmol Na_z_MoO_4_ 2H_2_O, 0.04 μmol CoCl_2_ 6H_2_O, and 0.04 μmol CuSO_4_ 5H_2_O (Figure 10). Deionized water was employed as the substrate for the freshwater medium, whereas artificial seawater was prepared by dissolving 36.71 g L^−1^ Coral Pro Salt (Red Sea Fish Pharm Ltd., Eilat, Israel) in deionized water, resulting in a final salinity of approximately 34 ppt. At inoculation, the initial pH values of the freshwater and seawater media were 8.71 and 8.64, respectively. The culture period was 16 days. During the period, the strain was bubbled with CO_2_-enriched compressed air (1% CO_2_, *v*/*v*), filtered through a 0.2 μm sterile disposable filter that was provided with 200 μmol photons·m^−2^ s^−1^ one-side illumination by T8 fluorescent lamp (Philips, Eindhoven, The Netherlands) at 25 ± 1 °C.

### 4.2. Measurement of Microalgae Growth

The growth of the microalgal strain was evaluated through biomass concentration. Briefly, 10 mL of the culture was filtered through a pre-weighed 0.45 μm filter membrane (Changde Bkmam Biotechnology Co., Ltd., Changde, China). The retained biomass was sequentially rinsed with NaCl solutions of decreasing salinity (20, 10, and 5 ppt), followed by a final rinse with deionized water. The filters were dried at 80 °C for 24 h and re-weighed to a constant weight. The specific growth rate (μ) was calculated using the following equation [39]:

μ = (In X_2_ − In X_1_)/(t_2_ − t_1_) (1)

where X_1_ and X_2_ are the biomass concentration (g L^−1^) measured at two consecutive sampling times t_1_ and t_2_, respectively. The overall average specific growth rate (μ_avg_) was calculated over the entire cultivation period using the initial and final biomass concentrations. The maximum specific growth rate (μ_max_) was defined as the highest specific growth rate calculated between two successive sampling times.

### 4.3. Determination of Polysaccharide Content

The polysaccharide content was determined by the phenol–sulfuric acid method, as previously described [40]. The freeze-dried algal powder (10 mg) was added to 5 mL of 0.5 mol L^−1^ H_2_SO_4_ (Guangzhou Chemical Reagent Factory, Guangzhou, China), agitated at 80 °C for 0.5 h, and centrifuged at 8000 rpm for 10 min to obtain the supernatant. The extraction procedure was repeated three times, and the supernatants were subsequently combined and fixed to 50 mL.

### 4.4. Preparation of Polysaccharides

At the end of the cultivation period, the wet biomass was collected by filtration through a 600-mesh sieve, and the biomass powder was obtained by a vacuum freeze-dryer FD-1-50 (Biocool, Beijing, China). The freeze-dried algal powder was added to distilled water (1:20, *w*/*v*). The mixture was stirred in 80 °C for 4 h. The supernatant was then obtained by centrifugation at 8000 rpm for 10 min, which was the polysaccharide extract. After repeating the procedure three times, the extract was concentrated by RE-2000A vacuum evaporator (Yarong, Shanghai, China). Subsequently, the concentration was then precipitated by the addition of 4 times the volume of ethanol (100%) (Guangzhou Chemical Reagent Factory, Guangzhou, China) and then stored at 4 °C for 48 h. The precipitate was collected and the proteins were removed by the Sevag method [41]. The aqueous phase was collected and dialyzed with distilled water for 48 h, followed by freeze drying to obtain polysaccharide powder.

### 4.5. Physicochemical Properties

#### 4.5.1. Scanning Electron Microscopy (SEM) Observation of Micro-Morphology

The microstructural characteristics of SPS were investigated by an Ultrahigh-Resolution Scanning Electron Microscope SU8600 (Hitachi, Tokyo, Japan) with an accelerating voltage of 3.0 kV. The samples were coated with gold, and then the morphological features were observed under high vacuum at ×10,000 and ×5000 magnifications [42].

#### 4.5.2. Molecular Weight Analysis

The molecular weight of SPS was determined by gel permeation chromatography (GPC). SPS were dissolved in distilled water configured to a final concentration of 0.1 mg mL^−1^ and filtered through a 0.45 μm microporous filter membrane. The solution was subsequently analyzed on a PL-GPC 50 Integrated GPC System equipped with two columns (300 × 7.5 mm, PL aquagel-OH Mixed-M PL 1149-6800 and PL 1149-6801) (Agilent, Santa Clara, CA, USA) on which the measurements were performed. The injection volume was 100 μL, maintained at a temperature of 40 °C, eluted with a 0.1 M NaNO_3_ solution at a flow rate of 1.0 mL min^−1^, and detected by a refractive index detector. The molecular weight was calculated using a standard curve of various dextran concentrations [43].

#### 4.5.3. Determination of Polysaccharide Chemical Composition

The total carbohydrate content was determined by the phenol–sulfuric acid method [40]. The protein content was determined by the Lowry method [44]. The concentration of glucuronic acid was measured by a colorimetric method based on a meta-hydroxyphenyl reagent [45]. The sulphate content was calculated in accordance with Grotjan [46]. The polysaccharide powder was treated with 2 mL of 1 mol L^−1^ HCl at 100 °C for 6 h. Following filtration through a 0.45 μm microporous membrane, the volume of distilled water was increased to 5 mL, and the sulfate content was quantified using a DIONEX ICS-2500 ion chromatograph (DIONEX, Sunnyvale, CA, USA).

#### 4.5.4. Determination of Monosaccharide Composition

The monosaccharide composition was determined by pre-column derivatization of a 1-phenyl-3-methyl-5-pyrazolone (PMP) reagent combined with high-performance liquid chromatography (HPLC) [47]. Samples were hydrolyzed with 2.0 mL of 2 mol L^−1^ trifluoroacetic acid (TFA) (Guangzhou Chemical Reagent Factory, Guangzhou, China) in sealed 10 mL glass ampoules under a nitrogen atmosphere at 120 °C for 4 h. Residual TFA was removed by co-evaporation with methanol under a stream of nitrogen, and the resulting residues were reconstituted in 2.0 mL of water. For PMP derivatization, 250 μL of 0.6 M NaOH and 500 μL of 0.4 M 1-phenyl-3-methyl-5-pyrazolone (PMP)–methanol were added, and the reaction was carried out at 70 °C for 1 h. Thereafter, 500 μL of 0.3 M HCl was added to neutralize the pH. Then, 1 mL carbon tetrachloride was added and mixed thoroughly, after which the samples were centrifuged at 2994× *g* for 10 min. The resulting supernatant was collected for HPLC analysis.

HPLC analysis was performed using a Shimadzu LC-20AD system (Shimadzu, Kyoto, Japan) with UV detection at 250 nm. The PMP derivatives were separated on an Xtimate C18 capillary column (4.6 mm × 200 mm × 5 μm) maintained at 30 °C. Isocratic separation was conducted using 0.05 mol L^−1^ potassium dihydrogen phosphate solution (pH 6.70, adjusted with NaOH) and acetonitrile (83:17, *v*/*v*) at a flow rate of 1.0 mL min^−1^. The injection volume was 20 μL. Individual monosaccharides were identified by comparing their retention times with those of the corresponding reference standards and quantified by the external standard method based on their chromatographic peak areas.

#### 4.5.5. Fourier-Transform Infrared (FT-IR) Spectroscopy Analysis

KBr (200 mg) was added to the samples and KBr pellets were prepared for FT-IR analysis. The infrared spectrum of SPS (400–4000 cm^−1^) was examined with an IR affinity-1 Fourier-transform infrared spectrometer (Shimadzu, Kyoto, Japan).

#### 4.5.6. Nuclear Magnetic Resonance Spectroscopy Analysis

SSPS and FSPS (10 mg) were dissolved in D_2_O (0.5 mL). The 1D-NMR (1H-NMR and 13C-NMR) and 2D-NMR (heteronuclear singular quantum correlation spectroscopy, HSQC) spectra of samples were acquired with a Bruker AVANCE HD 600 MHz NMR spectrometer (Bruker Daltonik GmbH, Leipzing, Germany).

### 4.6. In Vitro Antioxidant Activity Assay

SSPS and FSPS were dissolved in distilled water to prepare the solutions with a concentration of 0.5–2.5 mg mL^−1^. Ascorbic acid was chosen as a positive control to evaluate the scavenging activity of polysaccharides against a range of in vitro free radicals.

#### 4.6.1. DPPH (2,2-Diphenyl-1-picrylhydrazyl) Radical-Scavenging Activity

The DPPH scavenging activity of the samples was determined by referring to the method reported by Chen et al. [48] with appropriate modifications. Different concentrations of samples and 0.2 mL of aqueous ascorbic acid solution were added to an equal volume of 0.1 μM DPPH ethanol solution. The reaction was performed in the dark at 25 °C for 30 min, following which the absorbance was measured at 517 nm. The scavenging activity was calculated according to the following equation:

Scavenging activity (%) = (1 − (A_1_ − A_2_)/A_0_) × 100 (2)

where A_0_ is the absorbance of the control group (distilled water instead of the sample solution); A_1_ is the absorbance of the experimental group (sample solution); A_2_ is the absorbance of the blank group (ethanol instead of DPPH).

#### 4.6.2. ABTS (2,2′-Azinobis (3-Ethylbenzothiazoline-6-sulfonic Acid)) Radical-Scavenging Ability

The scavenging activity of the samples against ABTS radicals was determined according to the method described in [17]. Equal volumes of 7.4 mM ABTS diammonium salt solution and 2.6 mM potassium persulfate solution were mixed and diluted with phosphate buffer (pH = 7.4) until the absorbance at 734 nm reached approximately 0.70, which served as the working solution of ABTS radicals. The samples of different concentrations were added to 0.8 mL of ABTS free-radical working solution and subsequently placed in a water bath at 37 °C for 15 min in the dark. The absorbance was measured at 734 nm. The scavenging activity was calculated according to the following equation:

Scavenging activity (%) = (A_0_ − A_1_)/A_0_ × 100 (3)

where A_0_ represents the absorbance of the control group (distilled water instead of the sample solution), and A_1_ represents the absorbance of the experimental group (sample solution).

#### 4.6.3. Hydroxyl Radical-Scavenging Ability

The scavenging activity of the samples against hydroxyl radicals (-OH) was carried out according to Li et al. with minor modifications [49]. Equal volumes of 0.75 mM 1,10-phenanthroline, 0.75 mM FeSO_4_, and 0.15 M phosphate buffer (pH 7.4) were combined with different concentrations of samples, followed by the addition of an equal volume of 0.01% H_2_O_2_ in the final step of the reaction. The mixture was then agitated and reacted at 37 °C for 30 min, after which the absorbance was measured at 536 nm. The following equation was used to calculate the hydroxyl radical-scavenging activity:

Scavenging activity (%) = ((A_1_ − A_0_)/(A_2_ − A_0_)) × 100 (4)

where A_0_ represents the absorbance of the negative control group (distilled water rather than the sample solution), A_1_ represents the absorbance of the experimental group, and A_2_ represents the absorbance of the normal group (distilled water rather than hydrogen peroxide).

### 4.7. Statistical Analysis

All assays were repeated with three independent biological replicates and two technical replicates. Differences between the seawater and freshwater treatments were evaluated using two-tailed independent-samples *t*-tests in SPSS Statistics version 18.0. The IC_50_ (half-maximal inhibitory concentration) was determined using GraphPad Prism 10 to characterize the effective concentration of SPS when the scavenging activity of free radicals in the system reaches 50%. The results were considered significant at differences of *p* < 0.05 and presented as mean ± standard deviation.

## 5. Conclusions

Polysaccharides from *S. platensis* SCSIO-44012 H11 consisted mainly of α-configured glucans with trace sulfate and uronic acid residues. Cultivation in artificial seawater increased biomass and polysaccharide yields, yielding SSPS with a higher molecular weight, an additional β-anomeric signal, and lower sulfate and uronic acid levels compared to FSPS. Although both polysaccharides showed weak DPPH and ABTS scavenging activities, SSPS exhibited superior hydroxyl radical scavenging. These findings support artificial seawater cultivation as a strategy for increasing polysaccharide yield and enhancing the in vitro hydroxyl radical-scavenging activity of *S*. *platensis* H11 polysaccharides.

## Figures and Tables

**Table 1:** Biomass productivity and specific growth rate of *S. platensis* H11 cultured in seawater and freshwater.

	Average Specific Growth Rate (d^−1^)	Max Specific Growth Rate (d^−1^)	Biomass Productivity (g L^−1^ d^−1^)
Seawater	0.21 ± 0.03 ^a1^	0.61 ± 0.04 ^a2^	0.54 ± 0.05 ^a3^
Freshwater	0.20 ± 0.03 ^a1^	0.44 ± 0.03 ^b2^	0.46 ± 0.01 ^b3^

Different letters denote significant differences in the growth of *S. platensis* H11 in seawater and freshwater culture (^a1^: the average specific growth rate, ^a2,b2^: the max specific growth rate; ^a3,b3^: biomass productivity, *n* = 3; data: mean ± SD).

**Table 2:** Molecular weight of *Spirulina* polysaccharides.

	Mp (kDa)	Mw (kDa)	PD
SSPS	650.73	466.19	1.28
FSPS	490.92	276.97	2.36

Mp: molecular weight at the apex of the dominant chromatographic peak, Mw: weight-average molecular weight calculated by integration of the entire chromatographic distribution, PD (Mw/Mn): polydispersity, SSPS: seawater *Spirulina* polysaccharides, FSPS: freshwater *Spirulina* polysaccharides.

**Table 3:** Chemical and monosaccharide compositions of *Spirulina* polysaccharides.

		SSPS	FSPS
Chemical composition (% DW)			
Total carbohydrate		88.79 ± 0.80	83.34 ± 1.28 *
Protein		4.34 ± 0.27	6.69 ± 0.12 *
Uronic acid		4.92 ± 0.43	6.57 ± 0.29 *
Sulfate		0.56 ± 0.02	0.78 ± 0.04 *
Monosaccharide composition (% total monosaccharides)			
Predominant component	Glucose	95.12 ± 0.13	92.97 ± 0.14 *
Trace components	Glucuronic acid	2.49 ± 0.10	3.04 ± 0.11 *
	Rhamnose	1.19 ± 0.13	1.64 ± 0.12 *
	Galacturonic acid	0.37 ± 0.10	0.43 ± 0.10
	Fucose	0.29 ± 0.11	0.43 ± 0.11
	Mannose	0.20 ± 0.06	0.27 ± 0.07
	Galactose	0.08 ± 0.04	0.72 ± 0.09 *
	Xylose	0.17 ± 0.08	0.39 ± 0.09 *
	Arabinose	0.08 ± 0.03	0.11 ± 0.05

DW: dry weight, (SSPS: seawater *Spirulina* polysaccharides, FSPS: freshwater *Spirulina* polysaccharides). * Significant difference from SSPS: * *p* < 0.05. (*n* = 3, technical replicates; data: mean ± SD).

**Table 4:** IC_50_ of *Spirulina* polysaccharides for scavenging radicals.

IC_50_ (mg·mL^−1^)	DPPH	ABTS	OH
SSPS	4.10 ± 0.06 ^a1^	10.92 ± 0.16 ^a2^	2.53 ± 0.02 ^a3^
FSPS	4.43 ± 0.13 ^b1^	11.36 ± 0.02 ^b2^	3.07 ± 0.02 ^b3^
Vc	0.07 ± 0.02 ^c1^	0.01 ± 0.00 ^c2^	0.32 ± 0.01 ^c3^

SSPS: seawater *Spirulina* polysaccharides, FSPS: freshwater *Spirulina* polysaccharides, Vc: ascorbic acid was used as the positive control, DPPH: (2,2-Diphenyl-1-picrylhydrazyl radical, ABTS: 2,2′-azinobis (3-ethylbenzothiazoline-6-sulfonic acid radical, OH·: hydroxyl radical, IC_50_: half maximal inhibitory concentration. Different letters denote significant differences among the values of IC_50_ of different free-radical-scavenging activity of SSPS and FSPS (^a1–c1^: DPPH; ^a2–c2^: ABTS, ^a3–c3^: OH). Data are presented as mean ± SD (*n* = 3 biological replicates; technical duplicates were averaged). IC_50_ values > 2.5 mg mL^−1^ were extrapolated from fitted dose–response curves.

## Data Availability

The data presented in this study are available on request from the corresponding authors due to legal reasons.

## Abbreviations

The following abbreviations are used in this manuscript:

SPS	*Spirulina* polysaccharides
SSPS	Seawater *Spirulina* polysaccharides
FSPS	Freshwater *Spirulina* polysaccharides
GPC	Gel permeation chromatography
PMP	1-phenyl-3-methyl-5-pyrazolone
Mp	Molecular weight of the highest peak
Mw	Average molecular weight
PD	Polydispersity coefficient
HPLC	High-performance liquid chromatography
DW	Dry weight
FT-IR	Fourier-transform infrared
NMR	Nuclear magnetic resonance
HSQC	Heteronuclear singular quantum correlation spectroscopy
DPPH	2,2-Diphenyl-1-picrylhydrazyl
ABTS	2,2′-Azinobis (3-ethylbenzothiazoline-6-sulfonic acid)
OH·	Hydroxyl radical
IC_50_	Half-maximal inhibitory concentration
